# Clinical Longevity of Obturators in Patients with Jaw Defects: a Retrospective Cohort Study

**DOI:** 10.1007/s00784-024-05681-8

**Published:** 2024-04-30

**Authors:** Karina Zierden, Carolin Julia Koch, Bernd Wöstmann, Peter Rehmann

**Affiliations:** 1 Department of Prosthodontics, Dental Clinic, Justus-Liebig University, Schlangenzahl 14, 35392 Giessen, Germany; 2 Private Practice, Hessen, Germany

**Keywords:** Obturator, Maxillofacial prosthodontics, Aftercare, Kaplan–Meier, Survival

## Abstract

**Objectives:**

The primary objective of the present retrospective clinical study was to determine the survival time of obturators while analyzing possible influencing factors.

**Materials and methods:**

This retrospective clinical cohort study analyzed the influence of various clinical factors on the survival probability of obturators and their follow-up outcomes using Kaplan‒Meier analysis.

**Results:**

A total of 76 patients with 115 obturators were included in the study (47 men and 29 women, mean age 58.1 ± 18.1 years). The mean observation time was 3.0 ± 4.5 years (maximum 26.3 years). A total of 40.9% (47) of all obturators observed had to be replaced. The survival rate after 5 years was 79.5% for telescopic-crown-retained tooth-supported obturators, 86.9% for telescopic-crown-retained implant-supported obturators, 58.8% for removable full denture obturators, 22.1% for clasp-retained obturators and 0.0% for splints. The type of attachment, attendance at a regular follow-up and defect cause significantly influenced the survival of the obturators (p < .05).

**Conclusions:**

The findings obtained in this study support the recommendation of using implant-supported obturators. Telescopic-crown attachments, either tooth- or implant-supported, seem to be favorable in terms of survival time. Attendance at a strict follow-up program seems to have a major influence on the longevity of the obturators.

**Clinical relevance:**

The use of implant-supported obturators to cover permanent oral and maxillofacial defects is highly recommended. Additionally, the use of telescopic-crown attachments seems to be favorable in terms of survival time. Clasp-retained obturators and surgical splints should be used primarily for temporary restorations due to their shorter survival times.

## Introduction

The global increase in head and neck cancer cases in recent years has been well documented. In Europe, 128,600 new cases of lip, oral cavity or pharynx cancer were reported in 2020 [1]. The American Cancer Society estimated 54.540 new cases of oral cavity and pharynx cancer in 2023 for America alone [2].

The prosthodontic treatment of patients with temporary or permanent oral and maxillofacial defects due to tumors, cysts, injuries or congenital malformations is a special challenge for dentists. Affected patients have special needs regarding the rehabilitation of masticatory function, phonetics and aesthetics [3, 4]. Nevertheless, studies have shown high patient satisfaction with obturator restorations, especially with implant-supported obturators [4–7].

Obturators can be used as temporary restorations when immediate covering of the defect is not possible. However, obturators are used as permanent restorations when surgical coverage of the defect is not possible.

Depending on the oral situation (number and condition of remaining teeth, location and size of defect after surgery, xerostomia), obturators can be manufactured in different ways: as removable full dentures in patients with edentulism, as telescopic-crown or clasp retained dentures in patients with remaining teeth or dental implants or as surgical splints. Since there are no evidence-based, standardized or clear treatment guidelines, prosthetic rehabilitation is carried out using conventional techniques and methods.

In the current literature, no studies have evaluated the clinical performance of obturators with different attachments. Mostly, the focus of existing studies is on specific types of obturators, defects or their localization [8–10]. In particular, dental implants and implant-supported obturators have been observed [11–19].

## Objectives of the study

Therefore, the primary purpose of this retrospective study was to evaluate the clinical performance of different types of obturators and to calculate the survival times according to the presence of various clinical cofactors. Thus, possible modeling factors should be investigated for their influence on the clinical performance of obturators. In addition, the necessary first aftercare measures should be observed. Considering the limitations of the present study, the results obtained should contribute to determining the influence of various clinical factors on the success of treatment with obturator prostheses. Furthermore, the influencing factors identified can be used to develop evidence-based, standardized and clear treatment strategies and aftercare concepts for these patients.

## Materials and methods

### Study design and setting

In the context of the present retrospective cohort study, the data of patients who underwent surgery because of oral tumors, cysts or cleft palates and who presented at the Department of Prosthodontics, Justus Liebig University Giessen, Germany, for further prosthetic treatment were evaluated.

The following inclusion criteria were used: met the manufacturing and delivery criteria for an obturator at the department, had a minimum of one follow-up appointment at the department, and lacked surgical coverage of the defect. Accordingly, the following exclusion criteria were used: the obturator was manufactured and delivered outside our department, the patient never showed up after delivery of the obturator, and surgical coverage of the defect was performed.

In addition to the information collected for the covariates (see below), the following information was recorded for statistical analysis: date of delivery of the obturator (start point value); date of replacement or loss of function of the obturator or date of last visit at the department (target event); reason for loss of function/replacement of the obturator; and date and type of first aftercare measurement performed.

**Fig. 1:**
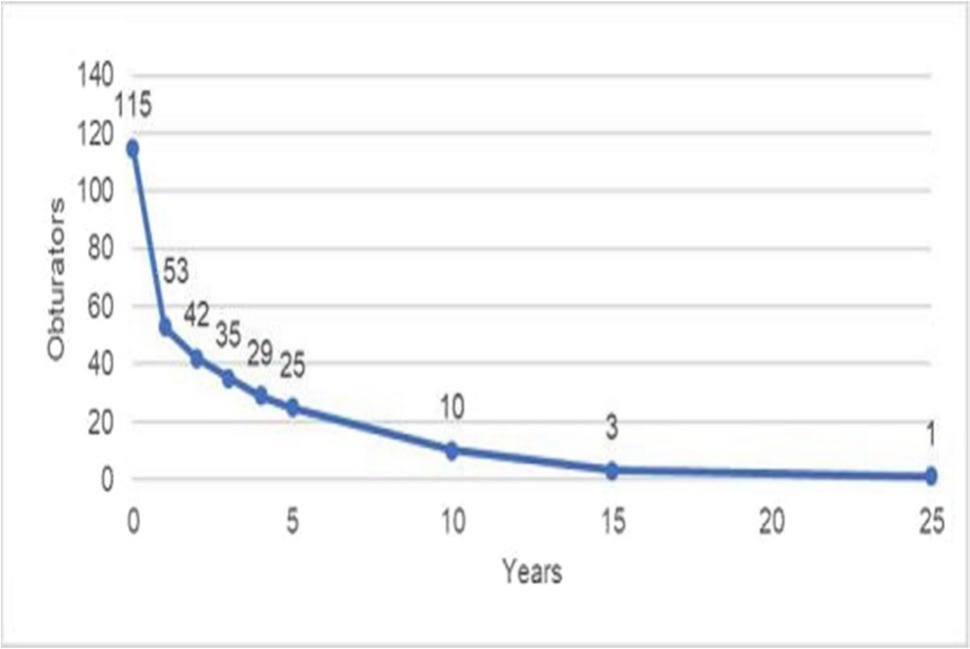
Number of obturators remaining at risk (n = 115)

The number of obturators remaining in the study by year is displayed in Fig. 1.

**Fig. 2:**
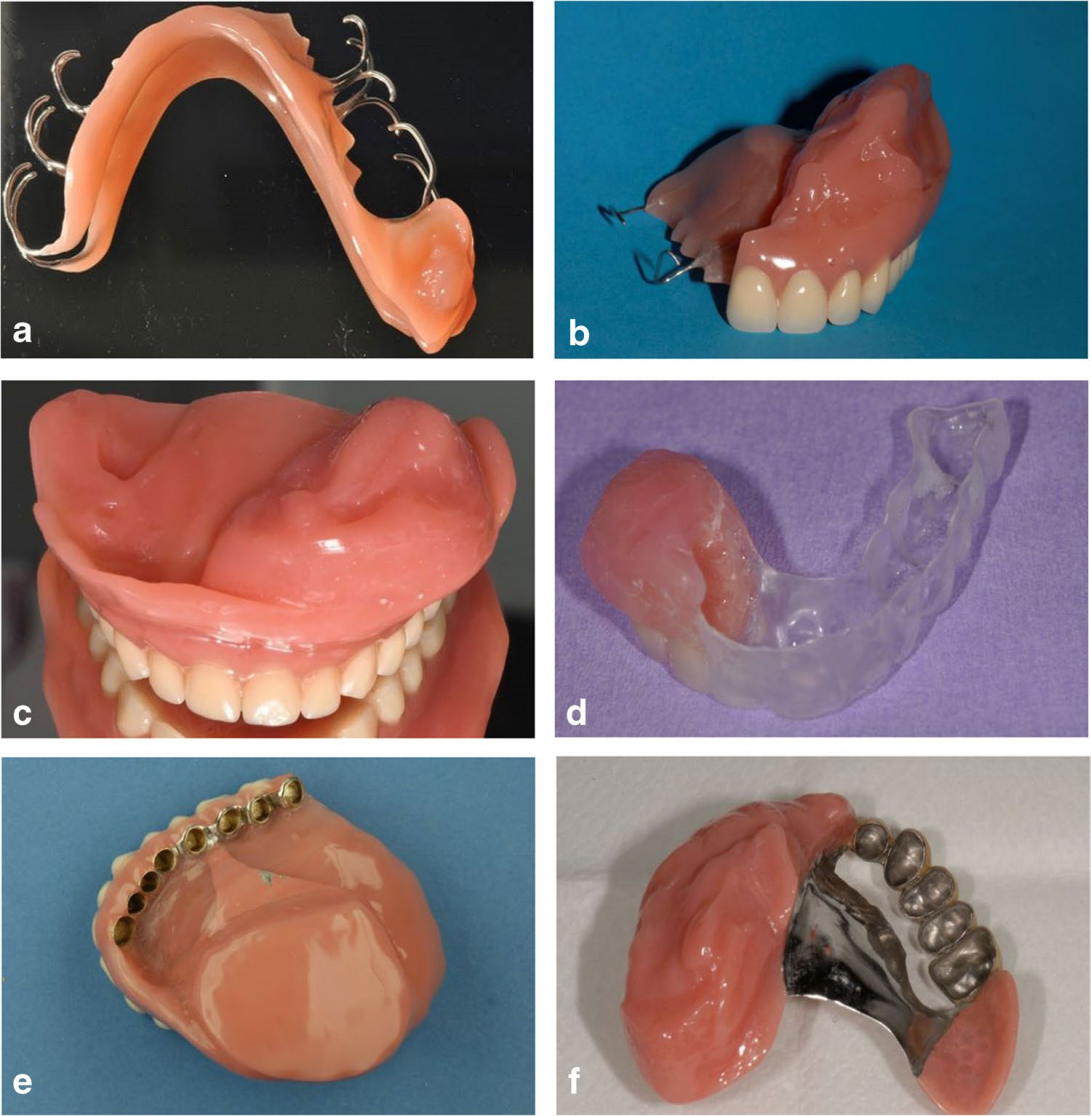
Different types of obturators observed in the study: **A**, clasp-retained obturator (mandible); **B**, clasp-retained obturator (maxilla); **C**, full denture obturator; **D**, occlusal splint; **E**, double-crown-retained implant-supported obturator; **F**, double-crown-retained tooth-supported obturator

Prosthetic treatment was performed by experienced dentists at the Department according to regular treatment procedures. Comprehensive planning, implant placement, and prosthetic rehabilitation were performed following an interdisciplinary protocol. An impression of the defect was taken intraoperatively to manufacture an interim obturator. The interim obturator was used immediately after removal of the tamponade. In the majority of cases, these were clasp retained (Fig. 2A, B) or removable full denture obturators (Fig. 2C) or, if the defect is small, surgical splints (Fig. 2D). The interim obturator was precisely adjusted as soon as wound healing was completed. Depending on the oral conditions of each patient (e.g., remaining teeth, dental implants), a permanent obturator was planned. Therefore, edentulous patients can be provided with either removable full denture obturators or, after placement of some dental implants, telescopic-crown-retained implant-supported obturators (Fig. 2E). Patients with adequate remaining teeth can be provided with telescopic-crown-retained tooth-supported obturators (Fig. 2F) or clasp-retained obturators. All telescopic-crown attachments used were parallel-sided. In some cases, if the interim obturator fits well and the patient can cope with it, the interim obturator will be adjusted and used as a permanent obturator. Treatment with permanent obturators follows the known treatment steps for regular prostheses, with special consideration of the obturator portion.

After delivery, the patients were enrolled in close monitoring with semiannual follow-up appointments. At this appointment, the condition, especially the leak tightness, of the obturator will be checked, and a full dental examination will be performed. In addition, oral hygiene measures were taken if necessary. Outside of these appointments, patients can present themselves immediately with any complaints.

### Statistical methods

The survival probability was assessed using Kaplan‒Meier analysis with 95% confidence intervals (CLs). The starting point value was set as the date of delivery of the obturator, and the ending point value was set as the date of replacement of the obturator. If the target event did not occur, the date of the last appointment at the department was set as the endpoint value. If several obturators were made for one patient, each obturator was considered a separate and independent case.

The significance level for the log-rank test to estimate differences between the individual covariates was determined at p = 0.05. Cox regression was also performed.

### Variables

The following variables were set and tested as possible influencing covariates on the survival of the obturators: patient sex (female or male or diverse); patient age at the time of delivery; type of attachment used (clasps or splints or full dentures or telescopic-crowns, either tooth-retained or implant-retained); location of the obturator (maxilla or mandible); cause of defect (malignant/benignant tumor or cysts or cleft palate); type of opposing dentition (fixed prostheses/natural teeth or removable prostheses or implant-supported prostheses or interim prostheses or full dentures or edentulous/no prostheses); and participation in a follow-up program (regular or only when problems occurred).

## Results

### Participants

In the first search, 133 patients were identified. According to the inclusion and exclusion criteria defined before 13 patients were excluded from the study because they had never attended a follow-up appointment after delivery of the obturator. 44 patients did not receive obturators after surgery for various reasons. Therefore, a collective of 76 patients with a total of 115 obturators were included in the study (47 men and 29 women, mean age 58.1 ± 18.1 years, maximum 87.9 years).

### Descriptive data

The mean observation time was 3.0 ± 4.5 years (maximum 26.3 years). 44 (38.2%) obturators were clasp retained, 25 (21.7%) obturators were telescopic-crown-retained implant-supported, 14 (12.2%) obturators were telescopic-crown-retained tooth-supported, 25 (21.7%) were removable full denture obturators, and 6 (5.2%) surgical splints were included. 97 prostheses were located in the maxilla, and 18 prostheses were located in the mandible. The exact distribution of the different types of obturators per jaw is shown in Table 1. A total of 104 (90.4%) obturators were manufactured for covering defects after malignant tumor surgery, five (4.4%) obturators were fabricated for covering defects after cyst removal, five (4.4%) obturators were manufactured for covering cleft palates, and one (0.8%) obturator was made for covering a defect following benign tumor surgery. 59 (51.3%) obturators were monitored regularly in the context of the annual follow-up program. 56 (48.7%) obturators could not be examined regularly since the patients were diagnosed only if they had problems. 68 obturators had natural teeth or fixed dental prostheses in the opposing jaw, 23 had removable dental prostheses (clasp or telescopic-crown retained), 20 had removable full dentures in the opposing jaw, two had implant-supported dental prostheses, one had an interim prosthesis in the opposing jaw, and one had an edentulous opposing jaw.

**Table 1:** Mean time (y) to loss of function/replacement of the obturator depending on localization and type of attachment

Type of attachment per Arch	Mean	SE	95% CL	
			Lower	Upper
Maxilla				
DCR* implant-supported obturators (n = 16, 2 lost)	7.50	1.03	5.47	9.54
DCR* tooth-supported obturators (n = 14, 5 lost))	15.09	3.03	9.15	21.04
Clasp retained obturators (n = 39, 24 lost)	2.16	0.44	1.28	3.04
Full denture obturators (n = 25, 6 lost)	8.79	1.55	5.74	11.84
Splints (n = 3, 1 lost)	0.67	0.00	0.67	0.67
Total (n = 97, 38 lost)	8.85	1.51	5.88	11.82
Mandible				
DCR* implant-supported obturators (n = 9, 3 lost)	9.85	2.17	5.58	14.11
DCR* tooth-supported obturators (n = 0)	-	-	-	-
Clasp retained obturators (n = 5, 3 lost)	0.45	0.15	0.14	0.76
Full denture obturators (n = 1, 1 lost)	2.60	0.00	2.60	2.60
Splints (n = 3, 2 lost)	2.91	1.52	0.00	5.89
Total (n = 18, 9 lost)	6.19	1.68	2.88	9.50

^*^DCR double-crown retained

### Main results

**Fig. 3:**
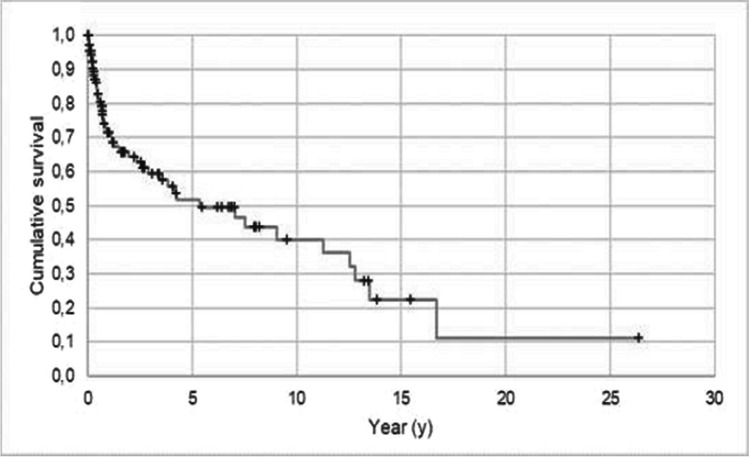
Outcome probability of all obturators (target event: loss of function/replacement; n = 115; Kaplan–Meier analysis). -, survival; + , censored

A total of 47 (40.9%) obturators had to be replaced. In 37 cases the interim obturator after tumor surgery was replaced with a permanent obturator (replacement of an interim obturator with a telescopic-crown retained or an implant-supported obturator). The reasons for replacement of the obturators are displayed in Table 2. The mean ± standard deviation (SD) expected survival time for obturators was 8.6 ± 1.4 years (95% CL: 5.8 to 11.3 years). After 5 and 10 years, 51.7% and 40.1%, respectively, of all the obturators were still functional (Fig. 3).

**Table 2:** Reasons for loss of function/replacement of the obturator

	n	%
Interim obturator changed into permanent obturator	22	46.8
Patient did not wear obturator after delivery	4	8.4
Subsequent coverage of the defect	3	6.4
Irreparable damage of the obturator	2	4.3
Implant loss	2	4.3
Loss of obturator	2	4.3
Tumor recurrence surgery	2	4.3
Other reasons	10	21.2
Total	47	100

**Fig. 4:**
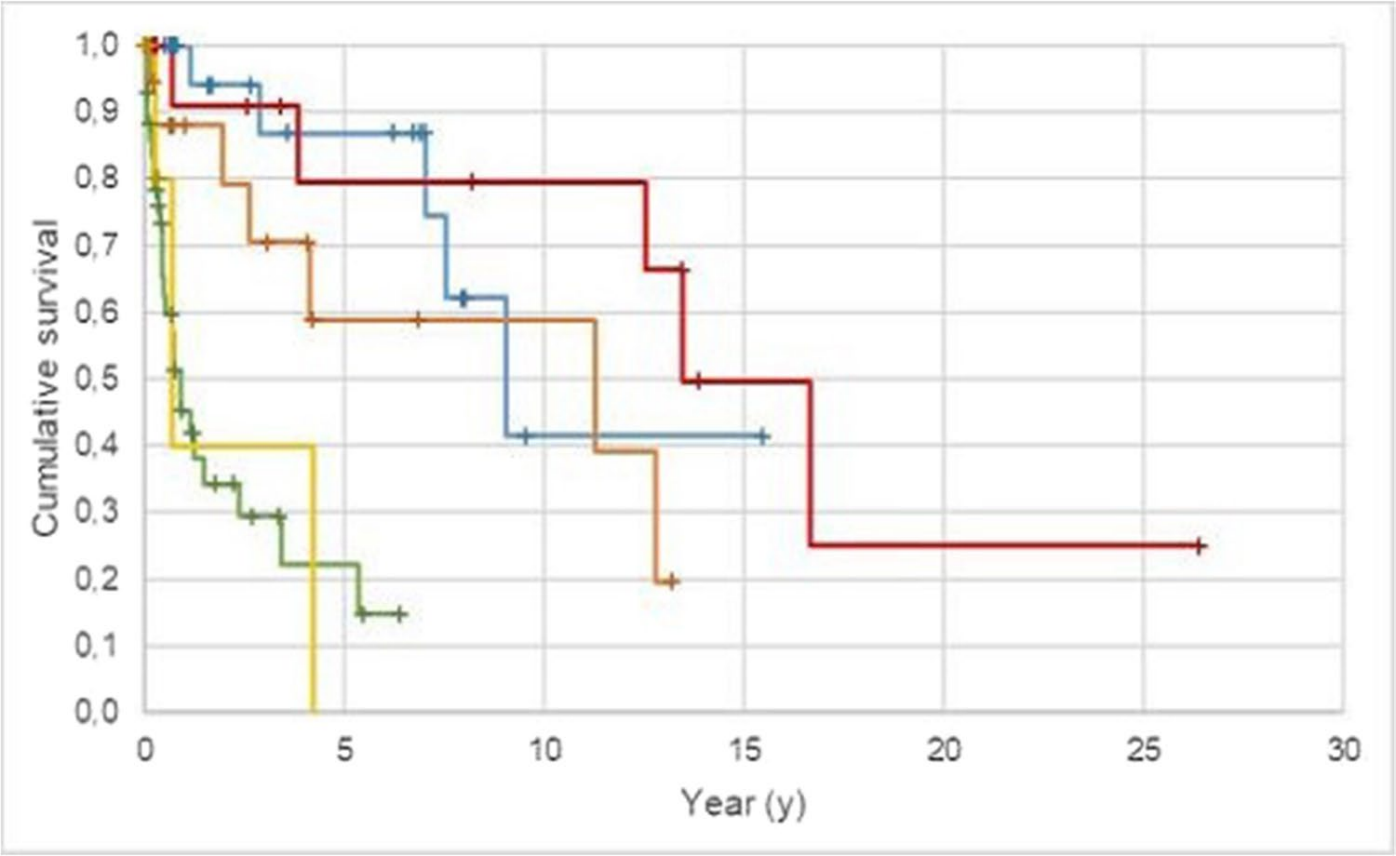
Probability of outcome for all obturators dependent on the attachment system (target event: loss of function/replacement; n = 115; Kaplan–Meier analysis).—DCR, implant-supported obturators;—DCR, tooth-supported obturators;—Clasp, retained obturators;—Full denture obturators;—Splints; + DCR, implant-supported obturators – censored; + DCR, tooth-supported obturators – censored; + Clasp, retained obturators – censored; + Full denture obturators – censored; + Splints – censored

The type of attachment used had a significant influence on the survival of the obturators (p < 0.05). Therefore, telescopic-crown-retained obturators had the best survival probability, followed by removable full-denture obturators, clasp-retained obturators and surgical splints (Fig. 4, Tables 3 and 4). The survival rates after 5 years were 79.5% for telescopic-crown-retained tooth-supported obturators, 86.9% for telescopic-crown-retained implant-supported obturators, 58.8% for removable full denture obturators, 22.1% for clasp-retained obturators and 0.0% for surgical splints. The ten-year survival rates were 79.5% for telescopic-crown-retained tooth-supported obturators, 41.1% for telescopic-crown-retained implant-supported obturators and 58.8% for removable full denture obturators. For clasp-retained obturators and surgical splints, the ten-year survival probability could not be estimated.

**Table 3:** Mean time (y) to loss of function/replacement of obturators depending on the type of attachment

Type of attachment	Mean	SE	95% CL	
			Lower	Upper
DCR* implant-supported obturators	10.345	1.628	7.155	13.535
DCR* tooth-supported obturators	15.097	3.034	9.150	21.044
Clasp retained obturators	2.069	0.426	1.233	2.904
Full denture obturators	8.212	1.529	5.215	11.209
Splints	2.019	1.321	0.000	4.609
Total	8.585	1.386	5.868	11.302

^*^DCR double-crown retained

**Table 4:** Five-/ten-year survival times and 90%/50% survival probability of obturators depending on the type of attachment

Type of attachment	Survival time %		Survival probability	
	5 years	10 years	90%	50%
DCR tooth-supported obturators	79.5	79.5	3.8 (y)	13.5 (y)
DCR implant-supported obturators	86.9	41.1	2.9 (y)	9.1 (y)
Full denture obturators	58.8	58.8	3.2 (m)	11.3 (y)
Clasp retained obturators	22.1	-	0.9 (m)	10.7 (m)
Splints	0.0	-	3.4 (m)	8.1 (m)

^*^DCR double-crown retained, *y* years, *m* month

**Fig. 5:**
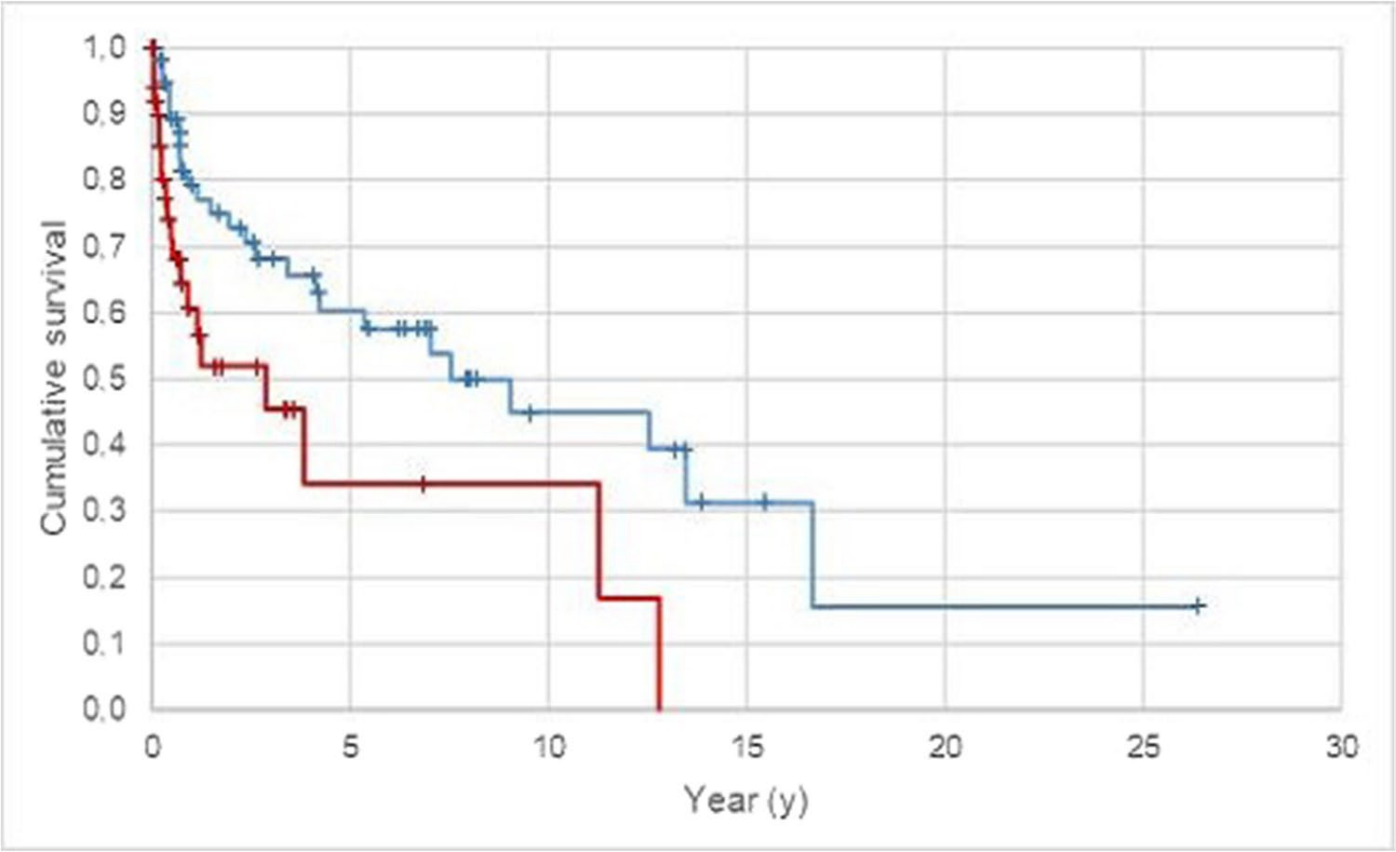
Probability of outcome for all obturators dependent on attendence in the follow-up program (target event: loss of function/replacement; n = 15, Kaplan–Meier analysis).—Attended regularly to follow-ups;—did not attend/only when they had problems; + Attended regularly to follow-ups – censored; + did not attend/only when having problems -censored

A significant influence on the survival probability of all types of obturators was observed for regular attendance at annual follow-up appointments (p < 0.05; Fig. 5). If the patient attended the annual follow-up appointment regularly, even without any problems, the mean survival time of the obturator was 10.3 ± 1.8 years (95% CL 6.7 ± 13.8 years). In comparison, obturators in patients who only appeared when having problems showed a significant lower survival time of 5.0 ± 1.2 years; 95% CL 1.2 ± 2.5 years). The 5- and 10-year survival rates for obturator patients who attended regularly in the annual follow-up program were 60.3% and 44.9%, respectively, whereas 34.1% for those who did not attend regularly.

**Fig. 6:**
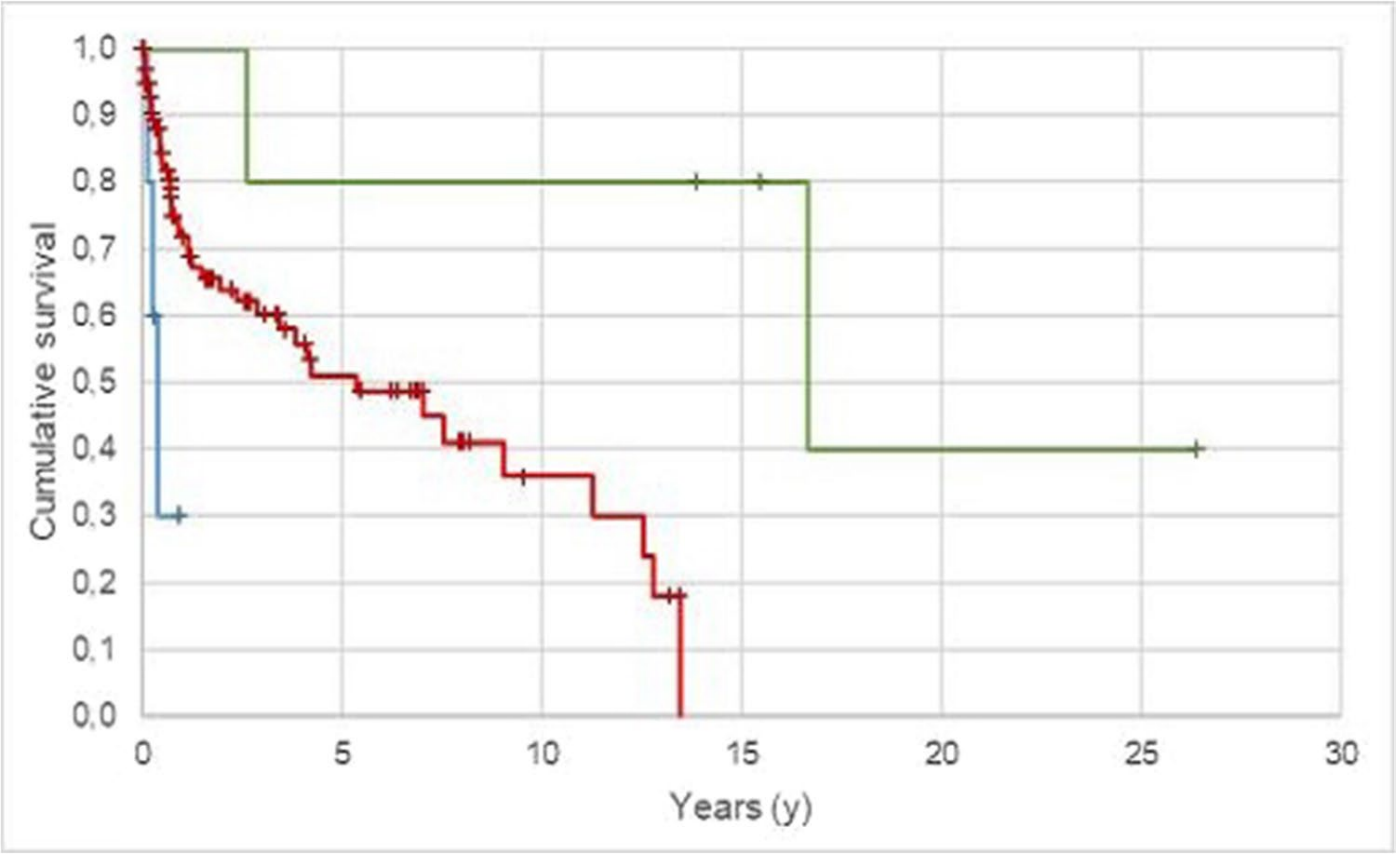
Probability of outcome for all obturators depending on the cause of the defect (target event: loss of function/replacement; n = 114; Kaplan–Meier analysis).—Cysts;—malignant tumors;—cleft palates; + cysts – censored; + malignant tumors – censored; + cleft palates – censored

Additionally, the cause of the defect had a significant influence on the survival of the obturators (p < 0.05). Obturators covering defects after cyst removal had the lowest survival times, followed by those whose defects were covered after malignant tumor removal and cleft palate (Fig. 6, Tables 5 and 6). Because of the small sample size, defects after benign tumor surgery (n = 1) were not included in this calculation.

**Table 5:** Mean time (y) to loss of function/replacement of the obturators depending on the cause of the defect

Cause of defect	Mean	SE	95% CL	
			Lower	Upper
Cysts	0.489	0.150	0.194	0.784
Malignant tumor	6.451	0.730	5.019	7.882
Cleft palate	17.739	4.355	9.203	26.274
Total	8.585	1.386	5.868	11.302

**Table 6:** Five-/ten-year survival times and 90%/50% survival probability of the obturators depending on the cause of defect

Cause of defect	Survival time %		Survival probability	
	5 years	10 years	90%	50%
Cysts	0.0	0.0	1.9 (m)	4.9 (m)
Malignant tumor	51.0	36.0	3.2 (m)	5.4 (y)
Cleft palate	80.0	80.0	2.6 (y)	16.7 (y)

*m* month, *y* years

Patient age at the time of incorporation showed a significant influence on the survival of the obturators (p > 0.05). A hazard ratio of 0.974 (95% CL: 0.959 to 0.990) showed that the risk of loss decreased by 2.6% if the patient age was increased by one year at the time of delivery.

The variables opposing dentition, patient sex and obturator location had no significant influence on the survival of the obturators (p > 0.05).

According to the Cox regression, only the type of attachment factor had a significant influence on the survival of the obturators. Therefore, clasp-retained obturators and splints were associated with a ten to six times greater risk of losing function than telescopic-crown-retained implant-supported obturators were (p > 0.05).

95 obturators needed at least one aftercare measure during the observation period. The mean time until the first aftercare was performed was 6.2 ± 1.5 months. The first necessary aftercare measure per obturator was distributed as follows: removal of pressure spots (n = 48, 63.2%), reline of the obturator base (n = 18, 23.7%), activation of clasps (n = 6, 7.9%), reattachment of primary crowns (n = 2, 2.6%), adjustment of acrylic parts (n = 2, 2.6%), and other parts (n = 15, 19.7%). All removable full dentures and splints observed needed at least one aftercare measure. A total of 87.1% of all clasp retained obturators, 85.7% of all tooth-supported telescopic-crown retained obturators and 79.2% of all implant-supported telescopic-crown retained obturators showed the need for aftercare.

## Discussion

### Key results

The primary objective of the present retrospective cohort study was to determine the survival time of obturators while analyzing influencing factors.

A total of 40.9% (n = 47) of all the obturators investigated lost their function or had to be replaced during the observation period. The cumulative five-year survival rate was 79.5% for telescopic-crown-retained tooth-supported obturators, 86.9% for telescopic-crown-retained implant-supported obturators, 58.8% for removable full denture obturators, 22.1% for clasp-retained obturators and 0.0% for surgical splints. The ten-year survival rate was 79.5% for telescopic-crown-retained tooth-supported obturators, 41.1% for telescopic-crown-retained implant-supported obturators and 58.8% for removable full denture obturators (clasp-retained obturators and splints did not reach ten-year survival). The type of attachment, attendance at a regular follow-up and defect cause significantly influenced the survival of the obturators (p < 0.05). The need for aftercare measures was high for all types of obturators in the present observation.

### Limitations

The present study had several limitations with regard to the patient population. Although the number of observed obturators in total (n = 115) was comparatively high, the individual groups were relatively smaller. In addition, the distributions of the different groups were unequal, as were the observation intervals. This made a direct comparison between the groups difficult. Although the study period spanned a total of 44 years, the average observation period was short, at 3.0 years (median 0.8 years). The short observation intervals could be justified by several factors. Many patients travel great distances to the clinic for surgery and postoperative prosthetic treatment. However, after prosthetic treatment at our department has been completed, in most cases, the family dentist takes over further care. Furthermore, some patients are in poor general condition, so follow-up visits at our department are not possible. Moreover, the mortality rate is knowingly high in these patients, so some of the patients die very soon. The cohort size was also rather small, at 76 patients. However, since head and neck tumors are rarer diseases and have a high mortality rate, a small sample size is the norm even for specialized facilities, as evidenced by the cohort sizes of other European studies [1, 2, 4]. Additionally, the study included only patients who underwent prosthetic treatment at our department following tumor surgery. This approach also reduced the patient population. A similar quality of prosthetic treatment and the manufacturing process of the obturators were noted in the present study. Moreover, the IT-supported documentation of the treatment processes within the Department of Prosthodontics can be tracked back to the exact date.

### Interpretation

There is no comparable study that specifically observed the survival time of obturators with different attachment systems. Most of the other studies dealing with patients suffering from temporary or permanent oral and maxillofacial defects have mainly involved implant-supported prostheses [3, 4, 11–19]. Moreover, some studies considered all types of prostheses (with and without obturators) or were limited to certain regions or defect causes [3, 8, 10, 14–18]. Weischer et al. observed 24 double-crown-retained implant-supported prostheses in patients with oral cancer and calculated a survival rate of 95% after nine years, which is considerably greater than our results (87%/41% after 5/10 years) [16]. Using quotient formation, Fukuda et al. calculated the 100% survival probability of implant-supported obturators in patients after partial or total maxillectomy [17]. However, the mean observation period was only 2.8 years, and the cohort consisted of seven patients only. A significantly lower survival probability was estimated for clasp-retained obturators in the present study (five-year survival probability 21%). This result is comparable to that of a previous study performed at the Department, which determined a five-year survival probability of 30% for clasp retained dentures among tumor patients [10]. These results might be explained by the fact that clasp retained obturators or dentures are mostly used as interim obturators or dentures and are not planned as permanent prostheses. Moreno et al. reported that moderate-sized maxillectomy defects can be treated successfully with either an obturator or free flap reconstruction [7]. Other studies have shown that edentulous patients treated with implant-supported obturators have a better quality of life [5, 6, 13, 17]. Genden et al. showed that patients who underwent palatomaxillary reconstruction achieved better mastication and speech function and less oronasal reflux than patients rehabilitated with prosthetic obturators [6].

The results of the present study showed no significantly greater survival probability for double-crown-retained implant-supported obturators than for telescopic-crown-retained tooth-supported obturators or full dentures.

Obturators covering defects after cyst removal (mean survival 4.9 ± 1.8 months) had the lowest survival time, followed by those with defects after malignant tumor (mean survival 6.45 ± 0.73 years) removal and cleft palate (mean survival 17.3 ± 4.3 years) removal. This result seems plausible because obturators for the treatment of cysts are intended only for temporary treatment. Murakami et al. observed three different types of obturators in patients with mandibular cysts or benign tumors with regard to their function and detected no major difference (mean survival 4.3–18.4 months) [9]. The mean survival time observed in the present study was 4.9 months for obturators covering defects after cyst removal, which is within the lower range of values analyzed by Murakami et al. [9].

If the patient regularly attended his follow-up appointments without any problems, the mean survival time for the obturators increased by 2.5 times. Regular follow-up appointments are necessary to detect possible decay at an early stage or to detect leaks or a bad fit of the obturators, for example. Comparable studies could not be found.

The need for aftercare measures was high in the present observation. This result is consistent with the findings from the current literature [18]. Clasp-retained obturators showed a 180% greater need for aftercare measures than did double-crown-retained implant-supported obturators in the present observation. This might be explained by the fact that clasp-retained obturators are mainly used as interim obturators and are delivered immediately after surgery. The manufacture of interim prostheses is usually more imprecise than that of permanent prostheses due to the use of different materials and methods. In addition, severe scarring occurs during the first few months after surgery, which requires constant adjustment of the interim obturator. Weischer et al. reported that only completely implant-supported prostheses avoided the occurrence of soft tissue ulcers, especially in patients after radiation [16]. This finding was confirmed in the present study.

## Conclusion

The findings obtained in this observation support the recommendation of the use of implant-supported obturators. Double-crown attachments, either tooth- or implant-supported, seem to be favorable in patients with maxillofacial defects in terms of survival time. If suitable residual dentition is present, double-crown-retained tooth-supported obturators are preferable to clasp-retained obturators. A longer survival time of the obturators, better wearing comfort, better protection of the teeth from possible damage by radiation through crowning, and easy expandability in cases of tooth removal are good reasons for extra effort.

The treatment of patients with acquired defects differs from that of patients with congenital defects.

The presence of cleft palate does not change significantly after growth is complete. In contrast, resection refers to an abrupt change in the physiology of the oral cavity and the processes that take place there. Prosthetic treatment should begin with good planning and preparation preoperatively to provide the patient with the best possible rehabilitation.

Patients should be motivated to participate in a strict follow-up program, as participation was found to have a major influence on the longevity of the obturators.

## Data Availability

No datasets were generated or analysed during the current study.

## References

[1] Dyba T, Randi G, Bray F, Martos C, Giusti F, Nicholson N (2021). The European cancer burden in 2020: Incidence and mortality estimates for 40 countries and 25 major cancers. Eur J Cancer.

[2] Siegel RL, Miller KD, Wagle NS, Jemal A (2023). Cancer statistics 2023. CA Cancer J Clin.

[3] Fierz J, Hallermann W, Mericske-Stern R (2013). Patients with oral tumors. Part 1: Prosthetic rehabilitation following tumor resection. Schweiz Monatsschr Zahnmed.

[4] Katsoulis J, Fierz J, IIzuka T, Mericske-Stern R (2013). Prosthetic rehabilitation, implant survival and quality of life 2 to 5 years after resection of oral tumors. Clin Implant Dent Relat Res.

[5] Buurman DJM, Speksnijder CM, Engelen BHBT, Kessler P (2020). Masticatory performance and oral health-related quality of life in edentulous maxillectomy patients: A cross-sectional study to compare implant-supported obturators and conventional obturators. Clin Oral Implants Res.

[6] Genden EM, Okay D, Stepp MT, Rezaee RP, Mojica JS, Buchbinder D (2003). Comparison of functional and quality-of-life outcomes in patients with and without palatomaxillary reconstruction: a preliminary report. Arch Otolaryngol Head Neck Surg.

[7] Moreno MA, Skoracki RJ, Hanna EY, Hanasono MM (2010). Microvascular free flap reconstruction versus palatal obturation for maxillectomy defects. Head Neck.

[8] De Vicente JC, Recio OR, Pendás SL, López-Arranz JS (2001). Oral squamous cell carcinoma of the mandibular region: A survival study. Head Neck.

[9] Murakami M, Nishi Y, Nishio M, Minemoto Y, Shimizu T, Nishimura MA (2019). Retrospective Cohort Study of the Cumulative Survival Rate of Obturator Prostheses for Marsupialisation. J Prosthodont.

[10] Zierden K, Wöstmann J, Wöstmann B, Rehmann P (2022). Clinical performance of different types of dental prosthesis in patients with head and neck tumors-a retrospective cohort study. Clin Oral Investig.

[11] Mericske-Stern R, Perren R, Raveh J (1999). Life table analysis and clinical evaluation of oral implants supporting prostheses after resection of malignant tumors. Int J Oral Maxillofac Implants.

[12] Buurman DJM, Speksnijder CM, de Groot RJ, Kessler P, Rieger JM (2020). Mastication in maxillectomy patients: A comparison between reconstructed maxillae and implant supported obturators: A cross-sectional study. J Oral Rehabil.

[13] Molinero-Mourelle P, Helm A, Cobo-Vázquez C, Lam WY, Azevedo L, Pow EH (2020). Treatment Outcomes of Implant-Supported Maxillary Obturator Prostheses in Patients with Maxillary Defects: A Systematic Review. Int J Prosthodont.

[14] Doll C, Nack C, Raguse JD, Stricker A, Duttenhoefer F, Nelson K (2015). Survival analysis of dental implants and implant-retained prostheses in oral cancer patients up to 20 years. Clin Oral Investig.

[15] Nelson K, Heberer S, Glatzer C (2007). Survival analysis and clinical evaluation of implant-retained prostheses in oral cancer resection patients over a mean follow-up period of 10 years. J Prosthet Dent.

[16] Weischer T, Mohr C (2001). Implant-Supported Mandibular Telescopic Prostheses in Oral Cancer Patients: An up to 9-Year Retrospective Study. Int J Prosthodont.

[17] Fukuda M, Takahashi T, Nagai H, Iino M (2004). Implant-supported edentulous maxillary obturators with milled bar attachments after maxillectomy. J Oral Maxillofac Surg.

[18] Laverty DP, Addison O, Newsum D, Bateman G (2023). Prosthodontic complications during implant-based oral rehabilitation of patients with head and neck cancer. J Prosthet Dent.

[19] Linsen SS, Martini M, Stark H (2012). Long-term results of endosteal implants following radical oral cancer surgery with and without adjuvant radiation therapy. Clin Implant Dent Relat Res.

